# Chemically Modified SDF-1α mRNA Promotes Random Flap Survival by Activating the SDF-1α/CXCR4 Axis in Rats

**DOI:** 10.3389/fcell.2021.623959

**Published:** 2021-02-04

**Authors:** Zucheng Luo, Yujie Bian, Gang Zheng, Huijing Wang, Bingqian Yan, Wenting Su, Wei Dong, Zhichao Hu, Jian Ding, Anyuan Wang, Shi Li, Wei Fu, Jixin Xue

**Affiliations:** ^1^Department of Orthopaedics, The Second Affiliated Hospital and Yuying Children's Hospital of Wenzhou Medical University, Wenzhou, China; ^2^Zhejiang Provincial Key Laboratory of Orthopaedics, Wenzhou Medical University, Wenzhou, China; ^3^The Second School of Medicine, Wenzhou Medical University, Wenzhou, China; ^4^Shanghai Children's Medical Center, School of Medicine, Institute of Pediatric Translational Medicine, Shanghai Jiao Tong University, Shanghai, China; ^5^Department of Dermatology, Wenzhou Hospital of Integrated Traditional Chinese and Western Medicine, Wenzhou, China; ^6^Department of Pediatric Cardiothoracic Surgery, Shanghai Children's Medical Center, School of Medicine, Shanghai Jiao Tong University, Shanghai, China; ^7^Shanghai Key Laboratory of Tissue Engineering, School of Medicine, Shanghai 9th People's Hospital, Shanghai Jiao Tong University, Shanghai, China

**Keywords:** SDF-1α, modRNA, SDF-1α/CXCR4, regeneration, random skin flap

## Abstract

Random skin flaps are frequently applied in plastic and reconstructive surgery for patients suffering from soft tissue defects caused by congenital deformities, trauma and tumor resection. However, ischemia and necrosis in distal parts of random skin flaps remains a common challenge that limits the clinical application of this procedure. Recently, chemically modified mRNA (modRNA) was found to have great therapeutic potential. Here, we explored the potential of fibroblasts engineered to express modified mRNAs encoding the stromal cell-derived factor-1α (SDF-1α) to improve vascularization and survival of therapeutic random skin flaps. Our study showed that fibroblasts pre-treated with SDF-1α modRNA have the potential to salvage ischemic skin flaps. Through a detailed analysis, we revealed that a fibroblast SDF-1α modRNA combinatorial treatment dramatically reduced tissue necrosis and significantly promoted neovascularization in random skin flaps compared to that in the control and vehicle groups. Moreover, SDF-1α modRNA transcription in fibroblasts promoted activation of the SDF-1α/CXCR4 pathway, with concomitant inactivation of the MEK/ERK, PI3K/AKT, and JAK2/STAT3 signaling pathways, indicating a possible correlation with cell proliferation and migration. Therefore, fibroblast-mediated SDF-1α modRNA expression represents a promising strategy for random skin flap regeneration.

## Introduction

Random skin flaps are frequently applied in plastic and reconstructive surgery (Glotzbach et al., 2010; Dehdashtian et al., 2019); however, the transplanted tissue sometimes fails due to poor blood supply in the distal region of the skin flaps, especially when the length-to-width ratio of the flap is >2:1 (Coskunfirat et al., 2014). The main factors that cause loss of the major vascular pedicles from the distal end of the flap, leading to inadequate blood perfusion of the skin flap, remain an enigma in plastic, and reconstructive surgery (Zhang et al., 2015). Over recent decades, researchers have trialed various methods to overcome these limitations by enhancing perfusion and blood flow to the tissue, including ischemic preconditioning, surgical delay and promotion of angiogenesis using exogenous proangiogenic growth factors, such as vascular endothelial growth factor (VEGF) (Xie et al., 2015; Akcal et al., 2016; Chehelcheraghi et al., 2019; Jiang et al., 2019) and stromal cell derived factor-1α (SDF-1α) (Song et al., 2018). SDF-1α, which belongs to the CXC chemokine family, has been studied extensively in relation to skin flaps. Indeed, in accordance with its well-known ability to promote neovascularization, SDF-1α overexpression was shown to stimulate angiogenesis and vasculogenesis (Yamaguchi et al., 2003).

Several studies have demonstrated the association of SDF-1α with flap vascularization and its ability to improve flap survival using approaches such as physical therapy, surgery and combined stem cell therapy. For example, Thanik et al. reported that low-dose radiation increased skin flap vascularity by augmenting angiogenic SDF-1α expression (Thanik et al., 2010). Carlo et al. demonstrated that escharotomy induced rapid endothelial progenitor cell (EPC) production and enhanced the release of SDF-1α into the circulation (Foresta et al., 2011). Zhang et al. reported that mesenchymal stem cells (MSCs) modified to express SDF-1α improved ischemic random flap survival (Zhang et al., 2011). However, the ability to achieve efficient expression and preserving the stability of SDF-1α in early interventions after flap transplantation remain a challenge (Liu et al., 2013).

Recently, chemically modified mRNA (modRNA) has been highlighted as a new approach in gene-based therapies. This technique can be used to achieve high-efficiency expression of virtually any protein without eliciting innate immune responses both *in vitro* and *in vivo* (Kariko et al., 2008). For example, intradermal injection of modRNA encoding VEGF-A has shown great potential for the treatment of diabetic wounds and cardiovascular disease (Sun et al., 2018). Recent studies also demonstrated that chemically modified VEGF-A mRNAs were well-tolerated and efficacy in the treatment of diabetic patients. ModRNA may hold promise as a regenerative treatment for patients with ischemic disease (Gan et al., 2019). Importantly, in our previous study, we demonstrated that cell-mediated modRNA delivery enhances the protein expression and promotes potent angiogenic effects, while decreasing the needed of modRNA (Yu et al., 2019).

In this study, we hypothesized that fibroblasts transfected with the modRNA encoding SDF-1α could better ameliorate ischemia and promote vascularization by high efficiency expression of SDF-1α leading to cell multiplication. *In vitro*, SDF-1α gene transfection effectively induced the expression of SDF-1α protein, which combined with CXCR4 (Ullah, 2019). In our study, we demonstrated that injection of modSDF-1α pre-treated-fibroblasts into the random skin flap significantly improved the survival area of the transplanted tissue *in vivo* by activating the SDF-1α/CXCR4 axis pathway. Our study provides valuable insights into a safe, novel, efficient, local, and controlled approach to improving the survival area of random skin flaps in the field of protein therapy.

## Materials and Methods

### Fibroblasts Isolation and Culture

Fibroblasts were obtained from abdominal skin of newborn rats as previously described (Lowe et al., 1990). Briefly, the skin was excised and washed with phosphate buffered saline (PBS) and disinfected with 70% ethanol for 10–30 s. The skin was then washed three time (~5 min per wash) with normal saline. Specimens were cut into pieces ~0.1 cm^2^ in size, and placed dermal-side down ~1 cm apart in a 50-ml culture flask using an elbow straw. The culture flask was turned so that the bottom of the bottle was facing upward incubated at 37°C for 2 h, allowing the skin specimens to dry slightly. The culture flask was then inverted and a small amount of Dulbecco's Modified Eagle Medium (DMEM) (Gibco, Invitrogen, Grand Island, NY) containing 10% fetal bovine serum (Hyclone, Thermo Scientific, Logan, UT, USA) and 1% antibiotics (Gibco, Invitrogen, Grand Island, NY) was added. The specimens were incubated at 37°C in a humidified atmosphere containing 5% CO_2_. The culture medium was changed once every 3 days, and the cells were subcultured for two generations. Finally, the fibroblasts were verified by hematoxylin & eosin (H&E) staining.

### ModRNA Synthesis and Formulation

Using a previously described (Richner et al., 2017), mRNA was synthesized *in vitro* using T7 RNA polymerase-mediated transcription from a linearized DNA template incorporating generic 5′-and 3′-UTRs and a poly-A tail. RNA was purified using Ambion MEGA clear spin columns and then treated with Antarctic Phosphatase (New England Biolabs) for 30 min at 37°C to remove residual 5′-phosphates. The purity and concentration of RNA were assessed using Thermo Scientific Nanodrop spectrophotometers. After purification, modRNA was resuspended at 1 μg/μl in 10 mM Tris HCl, 1 mM EDTA for use. In mRNA, uridine was fully replaced with N1-methylpseudouridine. The sequences of GFP and luciferase were as previously described (Zangi et al., 2013). The open reading frame sequence for SDF-1α modRNA as the follows:

ATGAACGCCAAGGTCGTGGTCGTGCTGGTCCTCGTGCTGACCGCGCTCTGCCTCAGCGACGGGAAGCCCGTCAGCCTGAGCTACAGATGCCCATGCCGATTCTTCGAAAGCCATGTTGCCAGAGCCAACGTCAAGCATCTCAAAATTCTCAACACTCCAAACTGTGCCCTTCAGATTGTAGCCCGGCTGAAGAACAACAACAGACAAGTGTGCATTGACCCGAAGCTAAAGTGGATTCAGGAGTACCTGGAGAAAGCTTTAAACAAGTAA.

### Fibroblast Transfection *in vitro*

Fibroblasts at passage two were transfected using Lipofectamine® Messenger MAXTM Reagent (Invitrogen, Life Technologies, USA) according to a previously described method (Yu et al., 2019). In brief, fibroblasts were cultured in 6-well plates at 2 × 10^5^ cells/well for 24 h at 37°C in a humidified atmosphere containing 5% CO_2_, and then cultured with fresh reduced-serum medium Opti-MEM (Gibco, Life Technologies, USA). ModRNA complexes were formed by mixing 2.5 μl Lipofectamine® MessengerMAX™ Reagent with 1 μg modRNA. Next, the density of cells required for transfection of fibroblasts at a dose of 10 pg/cell modRNA was calculated. That is, 2 μl of modRNA (1 μg/μl) was mixed with 48 μl Opti-MEM in tube A and incubated for 5 min, while 5 μl Lipofectamine was mixed with 45 μl Opti-MEM in tube B and incubated for 5 min. Subsequently, tubes A and tube B were mixed and incubated for 20 min at room temperature. The medium was then removed and the cells were washed twice with PBS before adding 1.5 ml fresh Opti-MEM to each well. For the SDF group, 100 μl of mixture containing 2 μg modRNA was added to each well. For the AMD group, cells were pre-treated with the CXCR4 antagonist AMD3100 (Sigma-Aldrich, St. Louis, USA) and an equal volume of mixture containing 2 μg modRNA was added to each well. For the Luc group, an equal volume of luciferase modRNA was added to each well. After transfection overnight, the medium was replaced with DMEM and cells were then cultured under standard conditions. GFP modRNA expression in fibroblasts was confirmed by photographing the cells at 2, 4, 12, and 24 h after transfection. The GFP modRNA transfection efficiency was measured by flow cytometry 24 h after transfection.

### Wound Healing Assay

The wound healing assay was used to assess the migration of fibroblasts after transfection with SDF-1α modRNA. Briefly, cells were seeded into the 6-well plates (1 × 10^5^ cells/well). At 100% confluence, a “wound” was created mechanically in a cell monolayer. The wound area was measured using ImageJ software (NIH, Bethesda, MD, United States) to quantify the migration rat of the cells with or without SDF-1α modRNA treatment after 0, 12, 24 and 48 h.

### Western Blot Analysis

Western blot analysis was performed as previously described (Luo et al., 2019b). Briefly, after homogenization, the total protein concentration was quantified using the BCA assay kit (Beyotime, Shanghai, China). Proteins (60 μg) from each sample was separated by SDS-PAGE and transferred to PVDF membranes (Bio-Rad, USA) that were blocked for 2 h using 5% non-fat milk. Subsequently, the membranes were probed overnight at 4°C with primary antibodies specific for GAPDH, SDF-1α, CXCR4, JAK2, p-JAK2, STAT3, p-STAT3, PI3K, p-PI3K, AKT, p-AKT, NF-kβ, MEK, and ERK (all were used at a 1:1,000 dilution). After washing, the membranes were then probed for 2 h at room temperature with appropriate secondary antibodies were visualized by enhanced chemiluminescence (Invitrogen) and protein bands were quantified with Image Lab 3.0 (Bio-Rad).

### Experimental Random Flap Model and Fibroblast Transplantation

**Figure 1:**
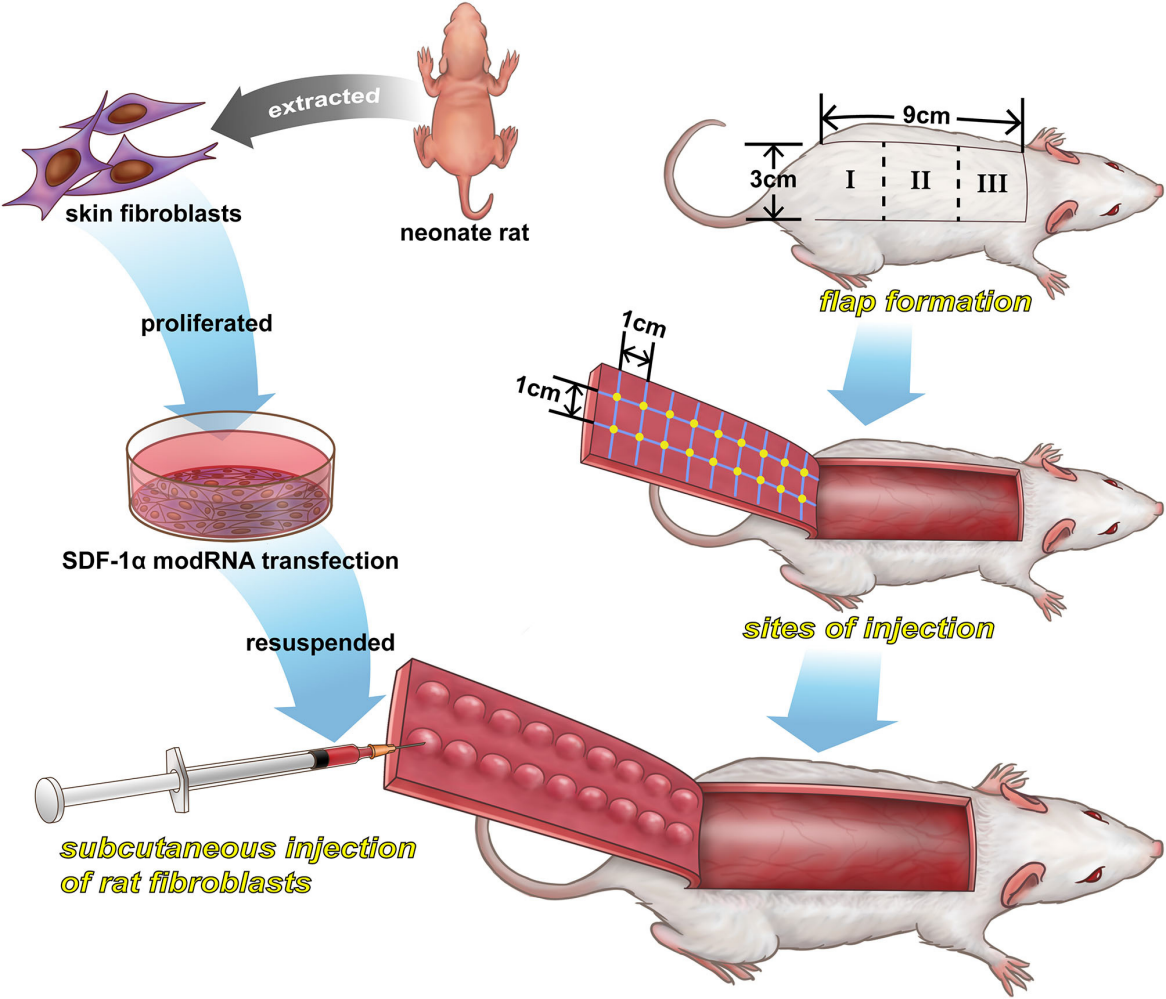
A schematic illustration of the preparation of SDF-1α modRNA-transfected in fibroblasts and the design of the *in vivo* experiments.

Sixty male Sprague-Dawley (SD) rats (250–300 g) were purchased from purchased from Animal Center of Chinese Academy of Sciences (Shanghai, China). The rats were randomized into control (PBS), luciferase modRNA (Luc) and SDF-1α modRNA (SDF) groups. The random flap model was established in the rat dorsum as previously described (Schweizer et al., 2013). Briefly, all animals were lightly anesthetized with isoflurane and then injected intraperitoneally with sodium pentobarbital (45 mg/kg). After shaving the hair, an area [3 cm (width) × 9 cm (length)] was marked as the random flap area on the back of the rat under aseptic conditions. The random skin flap was divided into three areas (area I, II, and III) as shown in Figure 1. Then, the full-thickness skin flap was elevated, the underlying fascia was removed, and any axial blood vessels entering the flap from the pedicle were excised. The PBS, Luc and SDF groups were injected with PBS, luciferase modRNA-transfected fibroblasts suspension and SDF-1α modRNA-transfected fibroblasts, respectively, at a cell density of 2 × 10^6^/cm^2^. The flap edge was bound, with 0.5 cm spacing, parallel to the flap of the length and width of the intersection, form a grid point injection, 0.1 mL solution was injected intradermally into each point of flap, a total of 18 injection points (Figure 1). Finally, the skin flap was sutured back to its normal position using 4–0 sutures (Ethicon, Johnson & Johnson Medical Products, New Jersey, USA). All rats were maintained individual cages with free access to food and water. This study was approved by the Animal Care and Use Committee of Wenzhou Medical University (ethics code: wydw2017-0159).

### Histology Analysis

The extracted skin flap specimens (*n* = 4) were paraffin-embedded and transversely cut into sections (5 μm thick). H&E staining was performed according to the manufacturer's instructions. Fibrotic accumulation was evaluated by Masson's trichrome staining according to standard procedures. Apoptosis was evaluated using a TUNEL cell apoptosis detection kit (Roche Applied Science, Indianapolis, IN, USA) according to the manufacturer's instructions.

For immunofluorescence and immunohistochemical staining, the sections were deparaffinized and rehydrated, followed by antigen-retrieval through incubation with trypsin for 30 min at 37°C. After blocking, the sections were stained with primary antibody overnight. For immunofluorescence analysis, specimens were incubated for 2 h at room temperature in dark with secondary antibody diluted in Antibody Dilution Buffer. After rinsing thoroughly with PBS, coverslips were added and the section were treated with anti-fade reagent and DAPI. For immunohistochemical analysis, goat anti-mouse HRP-conjugated was used as secondary antibody and the nuclei were stained with hematoxylin (Sigma) at the end of the procedures. Finally, the fluorescence intensity was quantified using ImageJ software.

Immunofluorescence cell staining was performed as previously described (Luo et al., 2019a). Briefly, sections were incubated with primary antibodies followed by DAPI staining to visualize the nucleus. The sections were viewed with a fluorescence microscope (Olympus, Tokyo, Japan) and fluorescence was quantified using ImageJ software.

### Enzyme-Linked Immunosorbent Assay

ELISA kits (R&D Systems, MN, USA) were used according to the manufacturer's instructions. *In vitro*, fibroblast culture supernatants were collected at various time-points (4, 8, 16, 24, 32, 40, 48, 72, 96, and 120 h) after transfection and the levels of SDF-1α were determined by ELISA. *In vivo*, the SDF-1α levels in the different flap areas (I, II, and III) were determined at various time-points (3, 7, 10, 14, 21, and 28 days) after surgery by ELISA.

### Skin Flap Survival and Blood Flow Assessment

The changes of skin flap were photographed for confirming the viable and necrotic parts of flaps in the same frame after surgery. The necrotic area of the skin flap was identified by the appearance of dark nidus, eschars, and hardening compared to the skin of normal rats. The area of flap survival was calculated with ImageJ software according to the following formula:

$$\begin{array}{l} \left(\text{Surviving area}÷\text{Total area}\right)\;\times \;100\text{\%}. \end{array}$$

The rats in each group were anesthetized and scanned using a Laser Doppler Imaging (LDI) system to evaluate the blood perfusion of the skin flap. The data were expressed as blood perfusion units (PUs).

### Tissue Edema

To measure the water content of the skin flap to evaluate the tissue edema, on day 10 after surgery, the specimens were weighed and dehydrated in an autoclave at 50°C, and weighed again for 3 days until a constant weight was achieved. The water content was calculated as follows:

([wet weight–dry weight] ÷wet weight) ×100%.

### qRT-PCR

Total RNA was extracted from cells in each group using TRIzol reagent (Invitrogen) according to the manufacturer's instructions. Total RNA from each sample was used to prepare cDNA by reverse-transcription (MBI Fermentas, Germany). The qRT-PCR was conducted using the following thermocycler settings were: 95°C for 10 min, followed by 40 cycles of 95°C for 15 s and 60°C for 1 min. A CFX96Real-Time PCR System (Bio-Rad, CA, USA) was used for all reactions. GAPDH expression was used to normalize all Ct values, and the 2^−ΔΔCt^ approach was used to calculate relative gene expression. The NCBI Primer-Blast Tool was used to design specific primers, which are listed in Table 1.

**Table 1:** Primers used in this study.

**Gene**	**Forward primer**	**Reverse primer**
SDF-1α	5′-TCTGCTCAGTGACGGTA-3′	5′-GGTACTCTTGGATCCACT-3′
CXCR4	5′-CTCTGAGGCGTTTGGTGCT-3′	5′-TGCCCACTATGCCAGTCAAG-3′
JAK2	5′-CAGATTCCGCAGGTTCATT-3′	5′-GTGGACGGTCACAACTCTACTT-3′
STAT3	5′-CGCCACTCTGGTGTTTCA-3′	5′-ATCTGCTGCTTCTCCGTCA-3′
PI3K	5′-TGCTATGCCTGCTCTGTAGTGGT-3′	5′-GTGTGACATTGAGGGAGTCGTTG-3′
AKT	5′-GTGCTGGAGGACAATGACTACGG-3′	5′-AGCAGCCCTGAAAGCAAGGA-3′

### Statistical Analysis

Data were presented as the mean ± standard deviation (SD). All statistical analyses were conducted with SPSS 20.0. One-way ANOVA with Tukey's test was used to compare control and treatment group data, while non-parametric results were compared using the Kruskal–Wallis *H*-test. *P* < 0.05 was considered to indicate statistical significance.

## Results

### ModRNA Was Efficiently Delivered in Fibroblasts With Protein Expression

**Figure 2:**
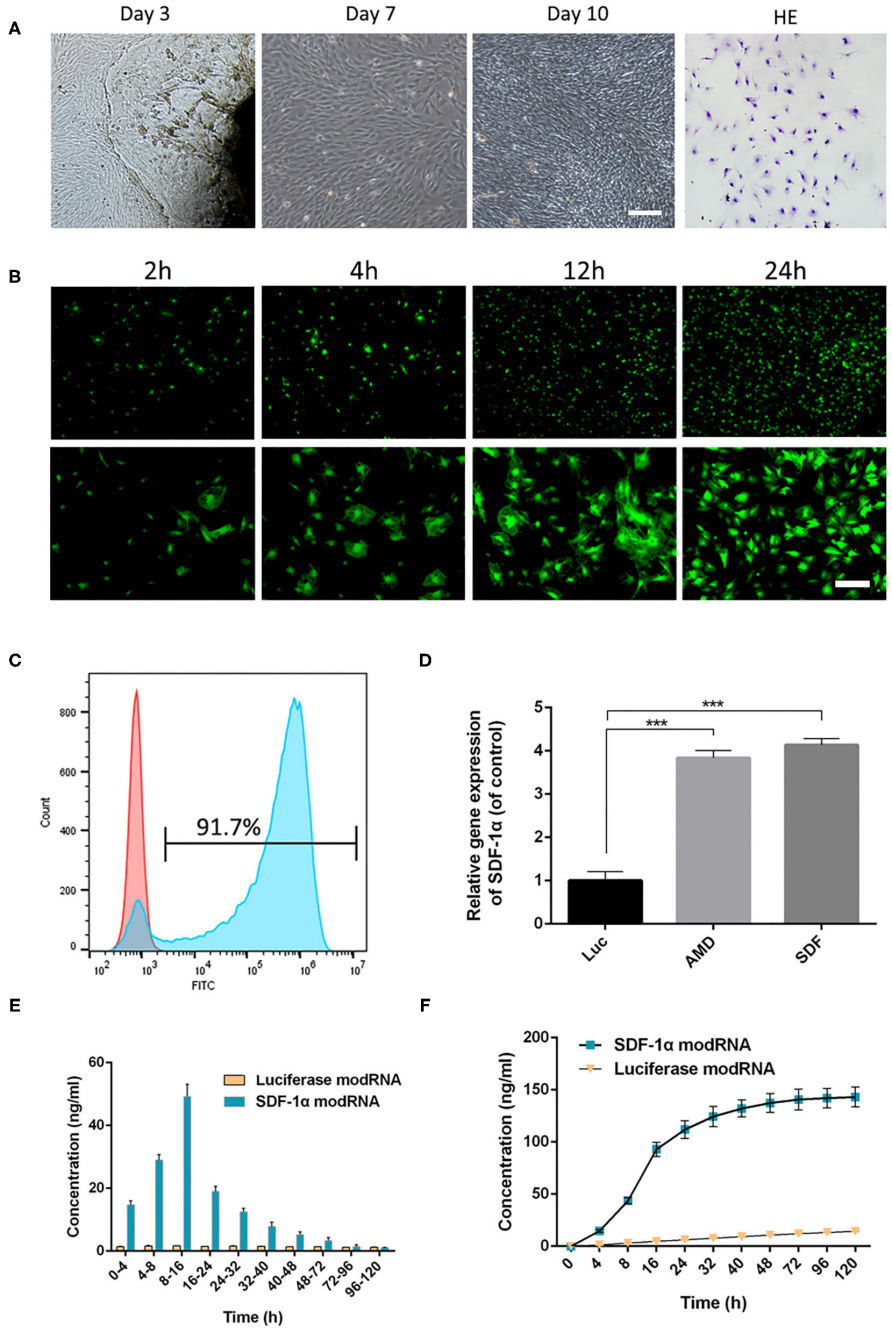
Skin fibroblasts were cultured and transfected with modRNA. **(A)** The fibroblasts were observed under light microscope and identified by H&E staining (scale bar: 50 μm). **(B)** Representative photo-micrographs depicting superior GFP signals in fibroblasts at 2, 4, 12, and 24 h post-transfection (scale bar: 50 μm). **(C)** Flow cytometric analysis of transfection efficiency 24 h post-transfection. **(D)** The expression of SDF-1α gene in fibroblasts was evaluated by qRT-PCR. **(E)** SDF-1α concentrations in culture supernatants of SDF-1α modRNA into fibroblasts. **(F)** Cumulative SDF-1α concentration after transfection of SDF-1α modRNA into fibroblasts. Data represent the mean ± SD (*n* = 5). ***P < 0.01.

First, the morphology of fibroblasts was observed under a light microscope. As shown in Figure 2A, fibroblasts were growing around the tissue block and adhered to the surface of the culture flask after 3 days. H&E staining showed typical fibroblast morphology, with a long spindle-like or irregular triangle structure. The number of cells was significantly increased by day 7 and some cells had grown to the middle of adjacent tissue blocks, while some regional cells were interconnected by day 10.

First, GFP modRNA was transfected into fibroblasts and the kinetics of protein expression were evaluated using fluorescence microscopy and flow cytometry. GFP modRNA was transfected into fibroblasts with high efficiency (92.9 ± 1.4%), and the intensity of the GFP protein signals increased gradually with time for 24 h (Figures 2B,C). In addition, qRT-PCR analysis of the levels of SDF-1α in SDF-1α modRNA-transfected with fibroblasts showed higher SDF-1α gene expression in the AMD and SDF-1α groups compared with those in the Luc groups (Figure 2D). To confirm that the modification produced higher levels of SDF-1α proteins the levels of SDF-1α protein were analyzed by ELISA immediately following transfection. SDF-1α concentrations were significantly higher after transfection with SDF-1α modRNA and SDF-1α concentrations reached a peak (49.24 ± 3.11 ng/ml) at 8 to 16 h followed by a gradual decrease, while SDF-1α expression was not detected at any time-point in cells transfected with luciferase modRNA (Figures 2E,F). These results confirmed that SDF-1α protein was highly expressed in fibroblasts transfected with SDF-1α modRNA.

### SDF-1α modRNA-Transfected Fibroblasts Activated the SDF-1α/CXCR4 Axis *in vitro*

**Figure 3:**
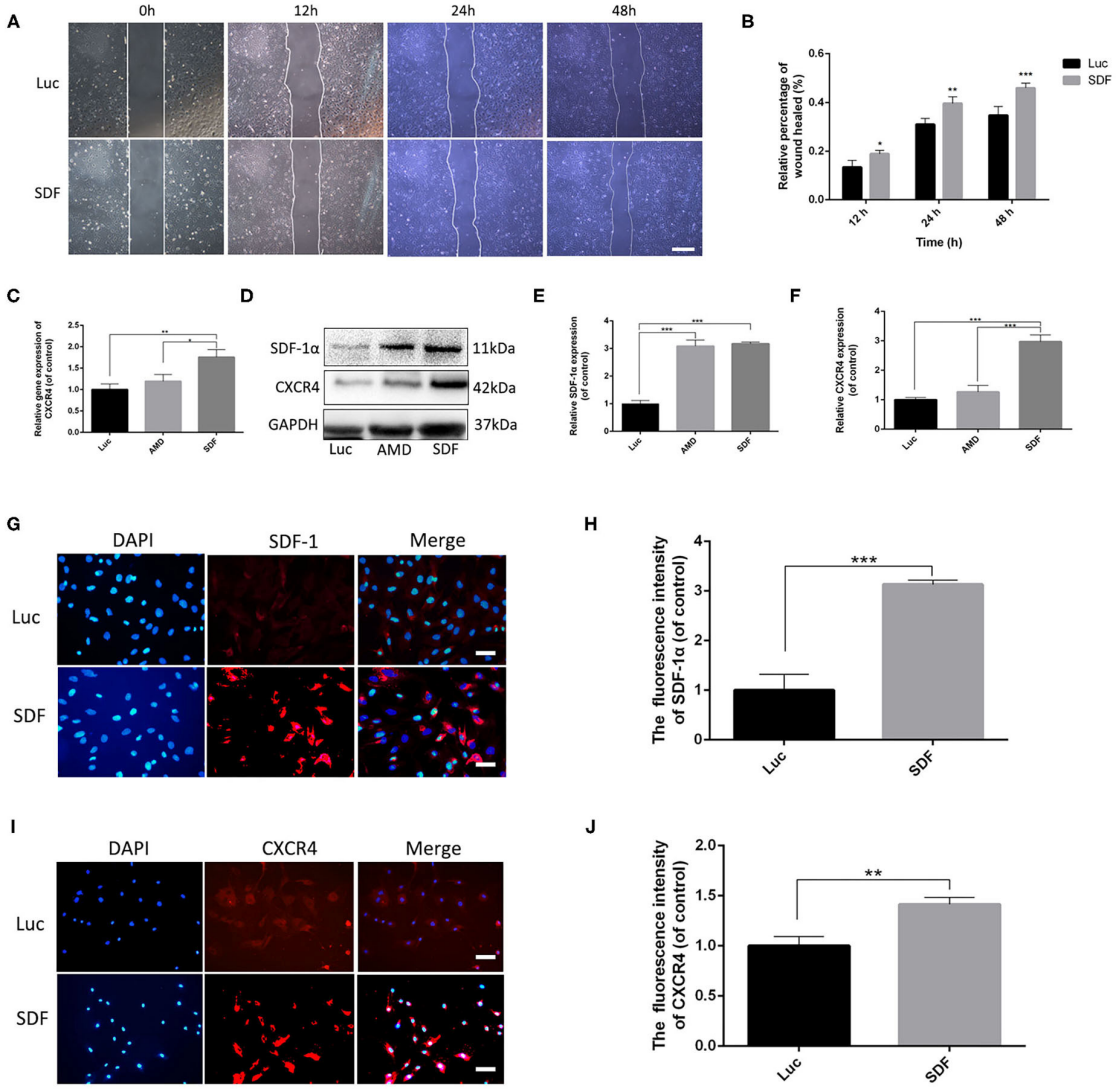
Fibroblasts transfected with SDF-1α modRNA promoted cell proliferation and activated the SDF-1α/CXCR4 axis *in vitro*. **(A)** Fibroblast migration evaluated using cell migration assays and **(B)** quantification (percentage) of gap closure at 48 h (bar: scale 50 μm). **(C)** CXCR4 gene expression was detected by qRT-PCR. **(D)** The expression of SDF-1α and CXCR4 proteins was evaluated by western blot analysis and quantified with Image Lab 3.0 **(E,F)**. Fluorescence analysis of the expression of SDF-1α **(G,H)** and CXCR4 **(I,J)** and quantified in each group using ImageJ software (scale bar: scale 50 μm). Data represent the mean ± SD (*n* = 5). *P < 0.05, **P = (0.01–0.05), ***P < 0.01.

To confirm that the SDF-1α protein secreted by fibroblasts transfected with SDF-1α modRNA was functionally active, we tested the ability of whether the conditioned medium to enhance the reproductive capacity of fibroblasts *in vitro*. The extent of wound healing was significantly impaired in the SDF group (Figures 3A,B). These findings suggested that SDF-1α mRNA promoted fibroblast metastasis. Several studies have indicated that the SDF-1α/CXCR4 signaling pathway is related to cell proliferation (Arjunan et al., 2018; Zhu et al., 2018). Consequently, to explore the roles of the SDF-1α/CXCR4 axis in fibroblasts after transfection with SDF-1α modRNA, we analyzed the expression of CXCR4 gene by qRT-PCR. CXCR4 expression was found to be significantly increased in the SDF group (Figure 3C). Moreover, we quantified CXCR4 protein levels in cell lysates 24 h post-transfection and found a significant increase above basal levels compared to those in the Luc groups (Figures 3D–F). In contrast, this pattern of CXCR4 expression was inhibited by the SDF-1α/CXCR4 antagonist (AMD3100) in the AMD group. Furthermore, immunofluorescence analysis of the SDF-1α/CXCR4 axis in fibroblasts revealed significantly increased SDF-1α and CXCR4 signals in the SDF group compared with those in the Luc group (Figures 3G–J). In summary, these results indicated that fibroblasts transfected with SDF-1α modRNA expressed SDF-1α at high levels and enhanced cell proliferation by activating the SDF-1α/CXCR4 signaling pathway *in vitro*.

### Transplantation of SDF-1α-Transfected Fibroblasts Attenuated Necrosis in the Skin Flap

**Figure 4:**
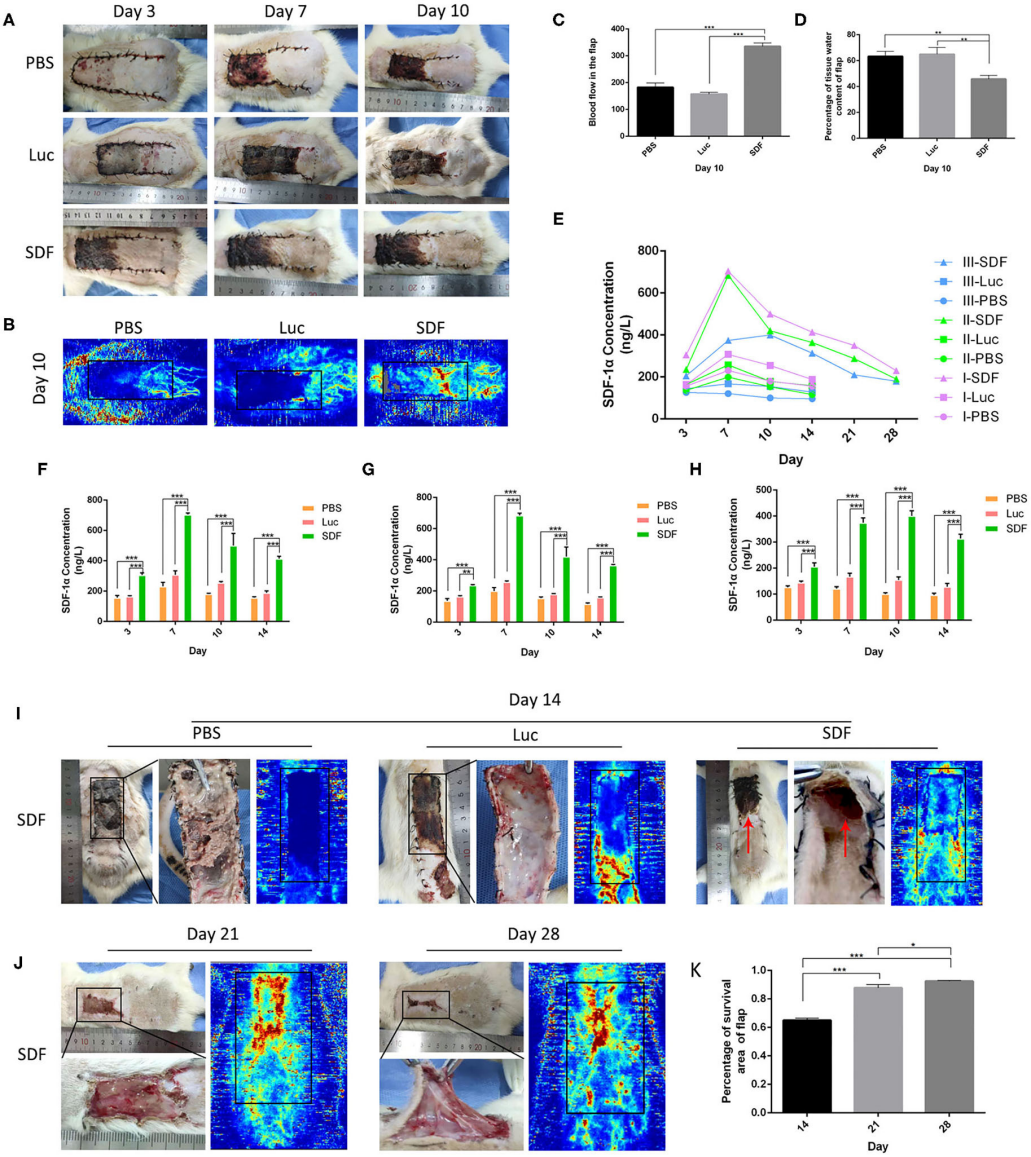
Flap survival area and blood perfusion in the rat random skin flap model. **(A,B)** Representative images of skin flap models at each time-point and LDI. **(C)** Histogram of percentages of flap survival area and **(D)** tissue water content of skin flaps on post-operative day 10. **(E)** Time-course of SDF-1α expression kinetics in random skin flaps post-transplantation, and are area of I, II, and III, respectively **(F–H)**. Representative images of skin flap models and LDI at day 14, 21, and 28 post-transplantation **(I,J)**. **(K)** Histogram of percentages of flap survival area. Data represent the mean ± SD (*n* = 4). *P < 0.05, **P = (0.01–0.05), ***P < 0.01.

We next examined the ability of fibroblasts transfected with SDF-1α modRNA to promote the survival of skin flaps *in vivo*. We transplanted SDF-1α or luciferase modified fibroblasts or PBS into the flap according to the method described in section Experimental Random Flap Model and Fibroblast Transplantation. The dorsal layer and necrosis in the distal part of the skin flap were assessed in photographs and using the LDI System at each time-point from day 3 to day 10. Representative photographs of the free skin flap in each group were shown in Figure 4A. Different degrees of necrosis at the dist4Eal end of the skin flap were observed in each group. A greater area of blood flow was observed in the SDF group compared with that in the PBS and Luc groups (Figures 4B,C). The water content of the tissue was significantly lower in the SDF group than that in the PBS and Luc groups (Figure 4D). Compared to the SDF groups, the skin flaps in the PBS and Luc groups were darker in color, rougher in texture and with more rigid surfaces on day 14. Subsequently, we evaluated the SDF-1α levels in the skin flap by ELISA. Compared with the other groups, higher levels of SDF-1α were detected in all three areas (I, II, and III) of the skin flap, with maximum SDF-1α levels observed in day 7 after treatment (Figures 4E–H). Consequently, we extended the observation time to 2 weeks. The rats were gradually died and there were more abscesses but no angiogenesis was observed at the distal end of the skin flap, possibly caused by distal necrosis and infection. LDI analysis showed poor blood flow in the PBS and Luc groups, while the blood flow was abundant in the skin flaps of the SDF groups. In contrast to the PBS and Luc groups, the skin flaps in the SDF group showed no significant changes in color and texture. Specifically, new skin was generated at the blackened and necrotic distal end of the skin flap (Figure 4I). When the observation time was extended to 4 weeks in the SDF group, the necrotic area of the distal end of the flap gradually healed and shrank with abundant angiogenesis under the distal end of the flap confirmed by the laser blood flow imaging (Figures 4J,K). Taken together these results confirmed the functional potential of fibroblasts modified to secrete SDF-1α protein for angiogenic therapy in random flaps *in vivo*.

### Transplantation of SDF-1α-Transfected Fibroblasts Promoted Blood Perfusion and Inhibited Apoptosis in Skin Flaps

**Figure 5:**
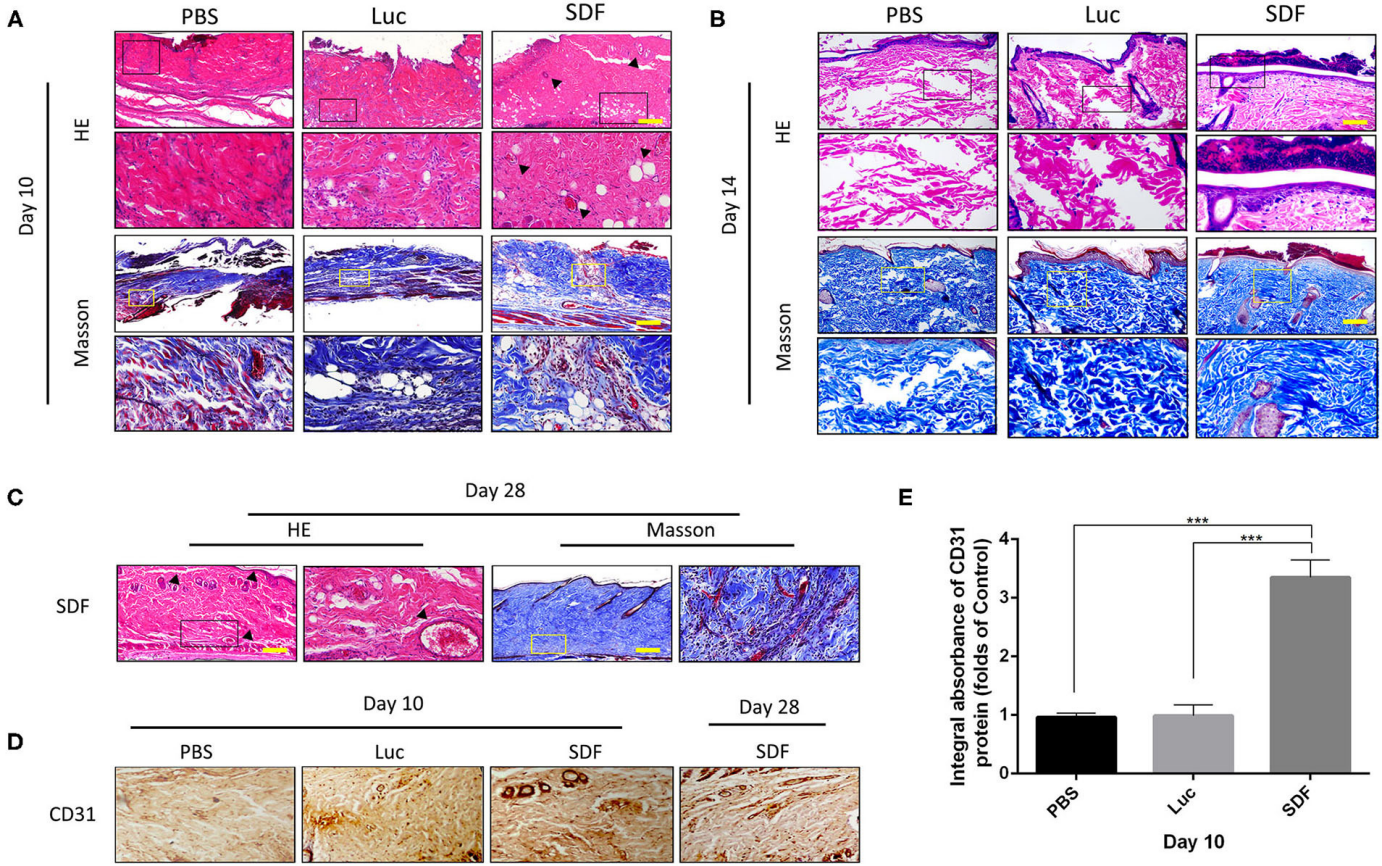
Histological evaluation of the skin flaps. **(A–C)** H&E and Masson's trichrome stained tissue sections from each group after 10, 14, and 28 days treatment. Black dotted square boxes indicate the location of microvessel (scale bar: 200 μm). **(D)** Immunohistochemical staining of CD31 expression in skin flaps of each group. **(E)** Quantification of immunohistochemical data. Data represent the mean ± SD (*n* = 4). ***P < 0.01.

**Figure 6:**
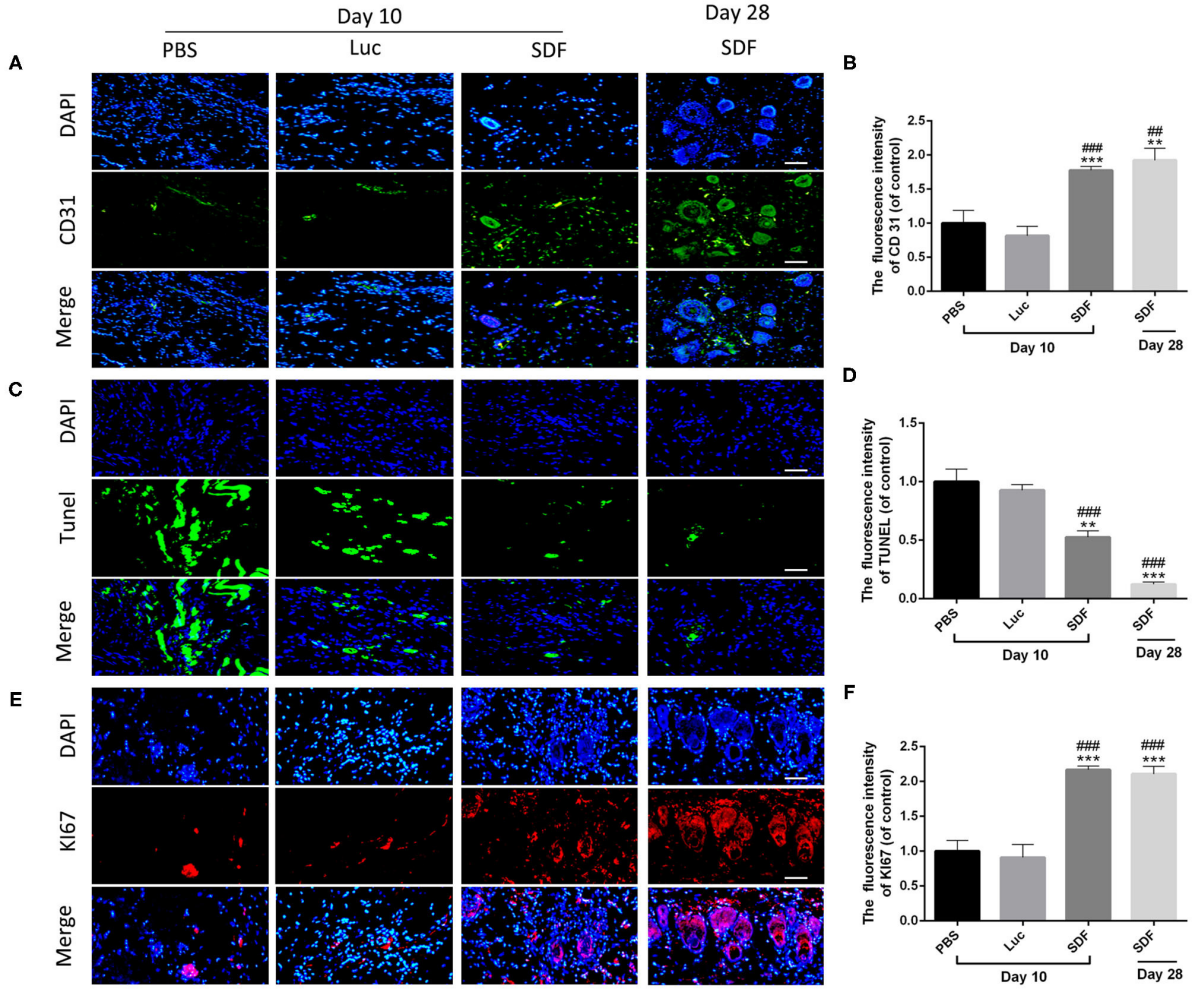
SDF-1α modRNA treatment promoted neovascularization and proliferation in the skin flap areas. Immunofluorescence staining of skin flaps of each group with **(A)** anti-CD31 antibodies (green) and DAPI (blue). **(B)** Quantification of immunohistochemical data (scale bar: 200 μm). **(C)** Cell apoptosis assessed by TUNEL staining. **(D)** Quantification of TUNEL staining data (scale bar: 200 μm). **(E)** Immunofluorescence staining of KI67 (red) and DAPI (blue). **(F)** Quantification of immunohistofluorescence data (bar: 200 μm). Data represent the mean ± SD (*n* = 4). **P = (0.01–0.05), ***P < 0.01 vs. PBS; ^*##*^*P* = (0.01–0.05), ^*###*^*P* < 0.01 vs. Luc.

Histological staining was performed to further evaluate the regeneration of the blood supply in random skin flaps. H&E staining showed that the skin flaps in the PBS and Luc groups were infiltrated by a large number of inflammatory cells with no obvious angiogenesis. In contrast, skin flaps in the SDF group contained numerous microvessels without severe inflammation at day 10. Masson's trichrome staining showed vascular infiltration and densely packed collagen fibers running in a parallel arrangement in the SDF groups, while the collagen fibers showed an irregular organization and were loosely packed in PBS and Luc groups (Figure 5A). At day 14, histological evaluation showed that collagen fiber assemblies were disorganized and fibrous connective tissue was disrupted in the PBS and Luc groups, while neoepidermis formation and marked vascularization was observed at day 28 post-surgery in the SDF group (Figures 5B,C). To further investigate the mechanism by which the SDF-1α modRNA transfected fibroblasts stimulated neovascularization, skin flaps were evaluated by immunohistochemical and immunofluorescent staining. Immunohistochemical analysis showed that SDF-1α modRNA transfection increased the number of CD31 positive vessels (Figures 5D,E), and the result was confirmed by immunofluorescent staining with an anti-CD31 antibody (Figures 6A,B). Furthermore, immunofluorescent analysis showed that cell proliferation was significantly promoted in skin flaps in the SDF-1α modRNA-transfected groups compared with that in the other two groups at day 28 post-surgery, while apoptosis was inhibited (Figures 6C–F). These results indicated that SDF-1α transfected treated fibroblasts secreted SDF-1α effectively and that these cells play a central role in the survival of random skin flaps.

### JAK-STAT, ERK, and PI3K–AKT Associated Genes Were Activated in modRNA-Transfected Fibroblasts *in vitro*

**Figure 7:**
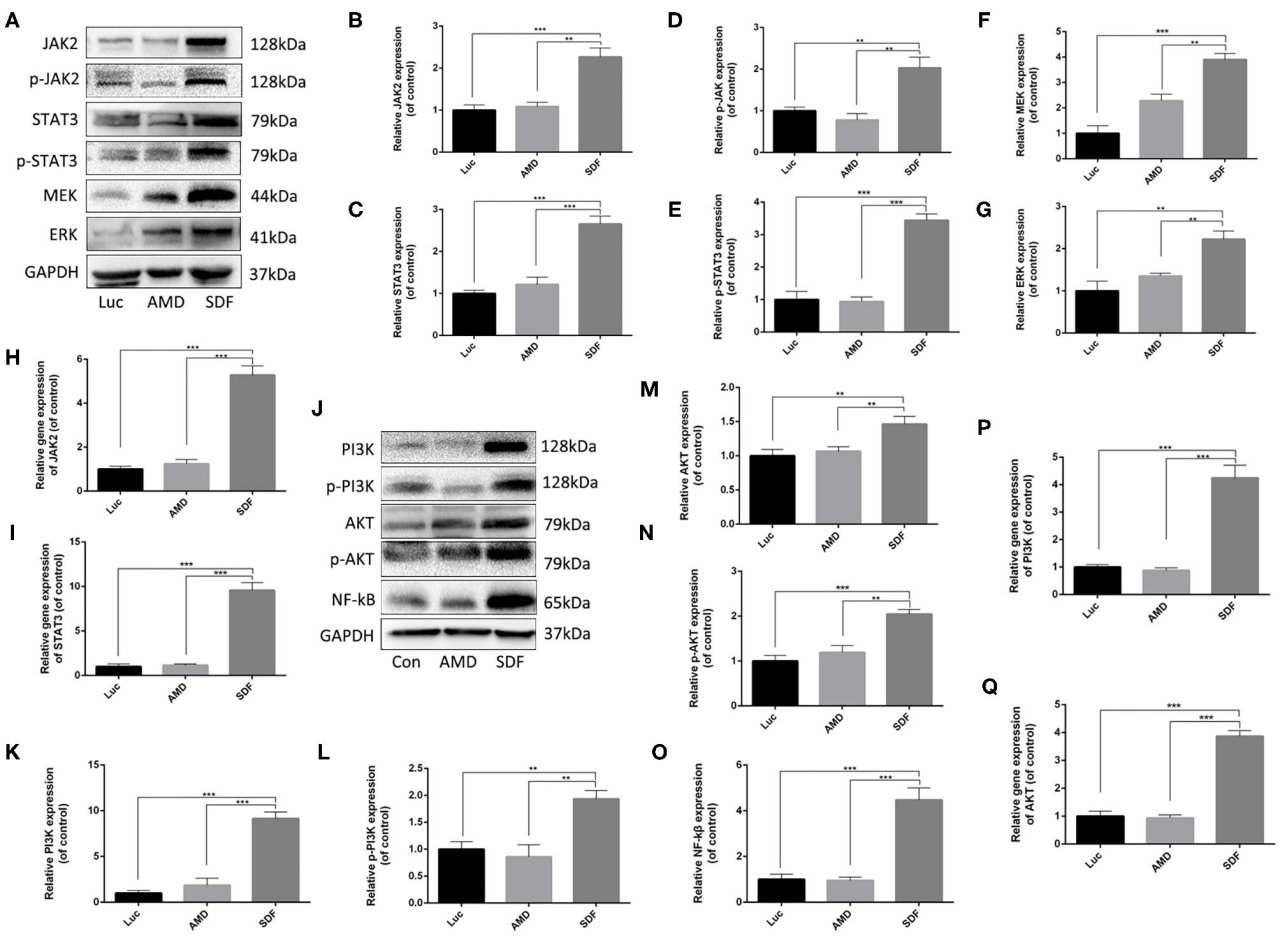
SDF-1α modRNA promoted cell proliferation and blocked apoptosis via activation of the JAK-STAT, ERK, and PI3K-AKT signaling pathways. **(A–G)** Representative western blots and quantification of JAK2, p-JAK2, STAT3, p-STAT3, MEK, and ERK proteins in fibroblasts after treatment with SDF-1α modRNA. **(H)** The expression of JAK2 and **(I)** STAT3 genes was detected by qRT-PCR. **(J–O)** Representative western blots and quantification of PI3K, p-PI3K, AKT, p-AKT and NF-kB proteins in fibroblasts after treatment with SDF-1α modRNA. **(P)** The expression of PI3K and **(Q)** AKT genes was detected by qRT-PCR. Data represent the mean ± SD (*n* = 5). **P = (0.01–0.05), ***P < 0.01.

**Figure 8:**
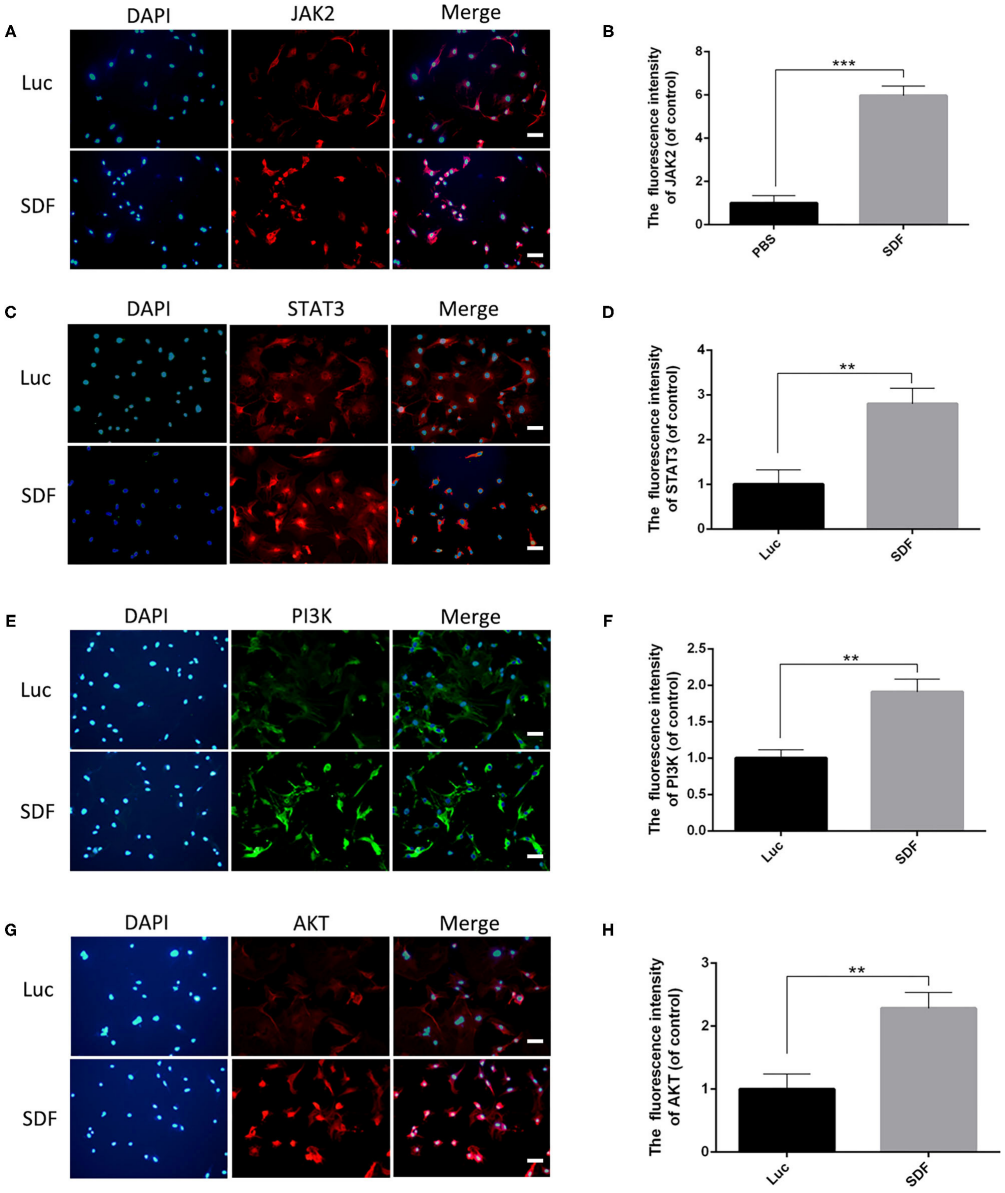
Immunofluorescence analysis of fibroblasts transfected with SDF-1α modRNA. Immunofluorescence staining with **(A)** anti-JAK2 antibodies (red) and DAPI (blue). **(B)** Quantification of immunohistofluorescence data (scale bar: 200 μm). **(C)** Immunofluorescence staining with anti-STAT3 antibodies (red) and DAPI (blue). **(D)** quantified in each group (bar: 200 μm). **(E)** Immunofluorescence staining with anti-PI3K antibodies (green) and DAPI (blue). **(F)** Quantification of immunohistofluorescence data (scale bar: 200 μm). **(G)** Immunofluorescence staining with anti-AKT antibodies (red) and DAPI (blue). **(H)** Quantification of immunohistofluorescence data (scale bar: 200 μm). Data represent the mean ± SD (*n* = 5). **P = (0.01–0.05), ***P < 0.01.

**Figure 9:**
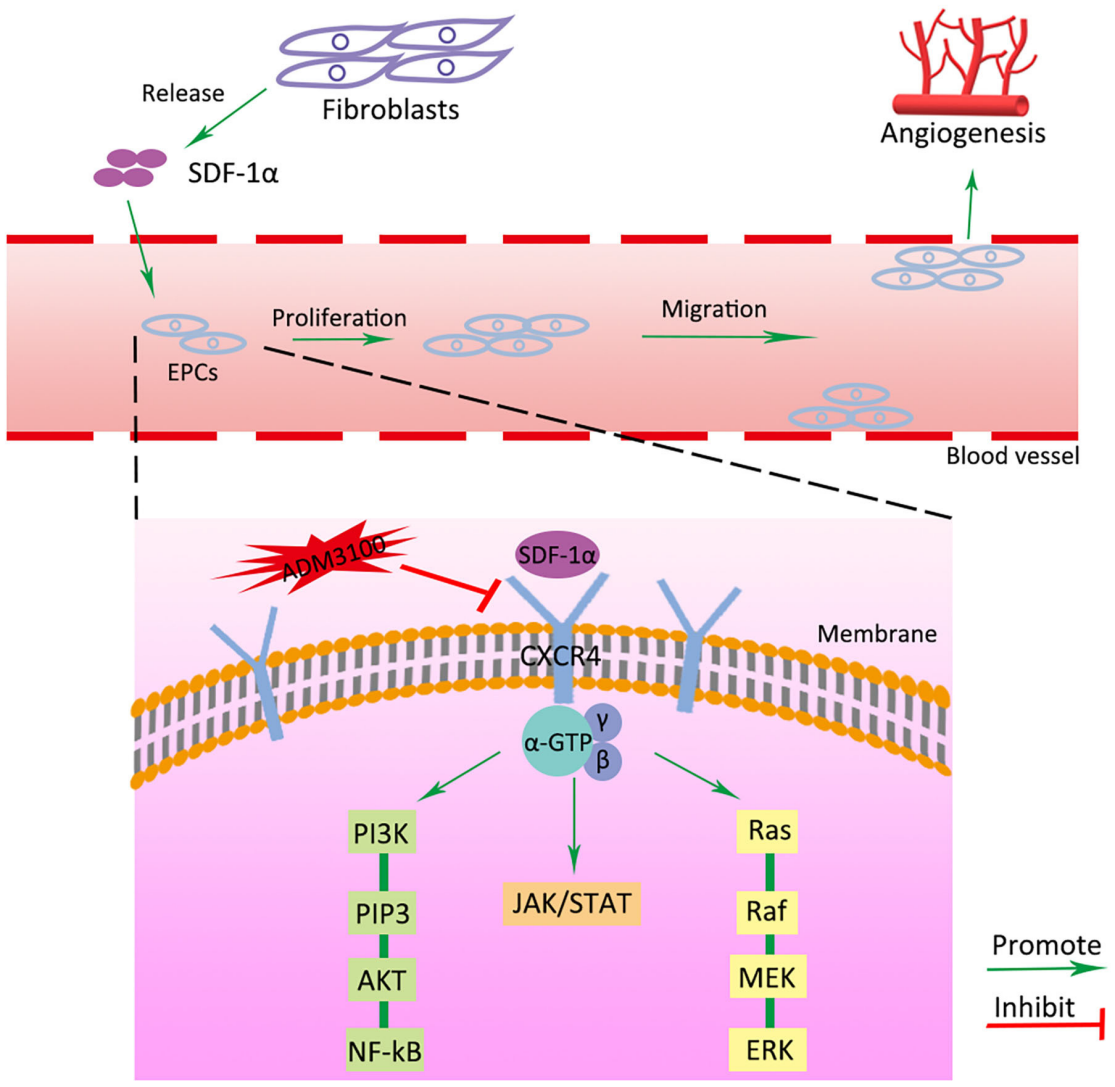
Schematic diagram of SDF-1α/CXCR4 signaling pathway activated by transfection of SDF-1α modRNA into fibroblasts. Green and red arrows correspond to positive and negative regulatory effects, respectively.

Previous studies have demonstrated that the CXCR4 axis activates the major physiological processes associated with cell invasion, migration and proliferation via a mechanism that involves the Janus kinase signal transducer and activator of transcription (JAK/STAT) pathway, the extracellular signal regulated kinases (ERK) pathway, and PI3K/AKT signaling pathway (Ding et al., 2019; Popielarczyk et al., 2019). We further explored the possible cellular mechanisms by which SDF-1α modRNA activated the SDF-1α/CXCR4 axis in fibroblasts. Compared to the Luc group, the SDF group exhibited a significantly increased ratio of JAK-STAT, MEK, and ERK at both the protein and gene expression levels assessed by western blot and qPCR, respectively (Figures 7A–I). Moreover, in comparison to the control group, the expression of PI3K, AKT, and NF-kβ was significantly increased in the SDF groups after transfection with SDF-1α modRNA (Figures 7J–Q). In accordance with this, immunofluorescence staining revealed higher expression of JAK2, STAT3, PI3K, and AKT in the SDF group than that in the Luc group (Figures 8A–H). Taken together, these results suggested that SDF-1α modRNA-transfected fibroblasts enhanced the proliferative capacity of cells via the MEK/ERK, PI3K/AKT, and JAK2/STAT3 signaling pathways (Figure 9).

## Discussion

Random skin flaps are routinely used in plastic surgery for reconstruction of large tissue defects as well as tissue loss caused by trauma, cancer and congenital disorders (Gurlek et al., 2004). However, ischemic necrosis is one of the most common complications in random skin flap surgery. In this study, we demonstrated that fibroblasts transfected with the modRNA encoding of SDF-1α expressed high levels of SDF-1α and promoted cell proliferation *in vitro*. Subsequently, we showed that SDF-1α modRNA transfected fibroblasts improve the survival area of random flaps *in vivo*. Furthermore, we revealed that the SDF-1α secreted by SDF-1α modRNA transfected fibroblasts combined with CXCR4 to and activate the SDF/CXCR signaling axis, stimulating activation of the downstream of MEK/ERK, PI3K/AKT, and JAK2/STAT3 signaling pathways to promote cell proliferation and inhibit apoptosis.

Nucleoside modification has gained much momentum as an effective approach to improve the translational capacity and stability of mRNA. This approach has numerous advantages including diminished its immunogenicity, safe local delivery and transient gene expression (Kariko et al., 2005; Anderson et al., 2010). A recent study demonstrated the safety, tolerability, efficacy, and functional outcomes resulting from intradermal delivery of modified VEGF-A mRNAs in diabetic patients (Gan et al., 2019). Importantly, we have previously demonstrated that transplantation of human dermal fibroblasts transfected with VEGF modified mRNA robustly promoted neoangiogenesis in ischemic tissue, indicating that this is a potential strategy for the treatment of critical limb ischemia (Yu et al., 2019). Hence, our previous study supports the feasibility of combining mRNA with cell therapy and/or tissue engineering strategies to achieve advances in the field of tissue regeneration.

Several previous studies have focused on the treatment of random flap necrosis with SDF-1α, using approaches such as combination with stem cell treatment, or SDF-1α gene modification of the skin flap (Liu et al., 2005; Yang et al., 2010; Cury et al., 2013; Zhou et al., 2019). It has been reported that local administration of SDF-1α reinforced vasculogenesis and promoted neovascularization *in vivo*, by inducing *in situ* recruitment of transplanted endothelial progenitor cells (EPCs) in ischemic tissues (Wils et al., 2017; Zhao et al., 2018). Transduction of mesenchymal stem cells with a recombinant adenovirus encoding the SDF-1α gene expressed high levels of SDF-1α, which increased neovascularization of early and partial ischemic free flaps and augmented the surviving areas. Thus, SDF-1α is closely associated with the survival of skin flaps. Therefore, the development of SDF-1α treatment strategies that are cost-effective will be of great importance in the fields of surgery and medicine.

Based on this evidence, we generated SDF-1α modRNA and further clarified the potential molecular mechanism by which SDF-1α regulates the SDF-1α/CXCR4 axis to promote the expression of vascularization-related genes at the mRNA and protein levels, demonstrating that these factors are key components required for microvessel and skin tissue regeneration (Eman et al., 2014; Pierscianek et al., 2017). In this study, we used terminally differentiated skin fibroblasts to deliver SDF-1α modRNA constructs driving high levels of SDF-1α expression to improve the survival of the distal end of skin flaps in a rat model. We observed partial necrosis of the distal flap in the SDF group at 10 days after surgery (Figure 4A), although microscopic blood vessels were generated under the skin flap (Figure 5A). Dramatically, new tissue and angiogenesis were found in the distal necrotic flap at day 14 post-surgery in the SDF group after delivery of fibroblasts transfected with SDF-1α modRNA. Interestingly, the maximum SDF-1α content was observed at day 7 after treatment (Figure 4E), although the SDF-1α content in the distal end of the flap in the SDF groups was still higher than that in the PBS and Luc groups at day 14. SDF-1α is highly expressed after trauma and ischemia and EPCs are known to migrate to sites of neovascularization in response to SDF-1α release, to contribute to wound healing (De Falco et al., 2004; Schonemeier et al., 2008; Li et al., 2015). In this study, a good wound surface without complete skin necrosis was observed in the SDF group after 7 days of treatment, combined with SDF-1α protein secretion, which recruited EPC to the skin wound, and promoted the wound healing. In addition, we speculated that the SDF-1α-transfected fibroblasts released large amounts of SDF-1α in the early stages, which may induce further release of SDF-1α by the flap tissue release resulting in more favorable conditions for wound repair. In addition, unlike VEGF, which strongly promotes angiogenesis, it is possible that SDF-1α induces recruitment of transplanted EPCs to enhance vascularization and also promote fibroblast proliferation to accelerate skin regeneration (Wang et al., 2010; Jin et al., 2013; Oh et al., 2019). However, the association of SDF-1α levels in the distal end of random skin flaps with vascularization and tissue formation requires further investigation. A comparison of the efficacy of SDF-1α modRNA and SDF-1α protein treatment or gene therapy of skin flaps is also required.

The results of this study demonstrate that fibroblasts transfected with SDF-1α modRNA express high levels of SDF-1α protein leading to activation of the SDF-1α/CXCR4 signaling pathways. This process promotes vascularization and tissue formation via the secretion of different chemokines, cytokines, and growth factors. Furthermore, injection of fibroblasts transfected with SDF-1α modRNA that secrete high levels of SDF-1α protein improved the survival area of random skin flaps *in vivo*. The safety and efficacy of this approach suggests that combining SDF-1α modRNA with fibroblast therapy represents a suitable platform for targeted protein therapies in random skin flaps.

## Data Availability Statement

The original contributions presented in the study are included in the article/supplementary material, further inquiries can be directed to the corresponding author/s.

## Ethics Statement

The animal study was reviewed and approved by Animal Care and Use Committee of Wenzhou Medical University (ethics code: wydw2017-0159).

## Author Contributions

JX and WF: conception and design, financial support, data analysis, and interpretation, and final approval of manuscript. ZL and YB: manuscript writing, collection, and assembly of data. GZ, HW, and BY: collection and assembly of data. WS and WD: provision of study material. ZH and JD: prepared figure. AW and SL: searched the literature. All authors contributed to the article and approved the submitted version.

## Conflict of Interest

The authors declare that the research was conducted in the absence of any commercial or financial relationships that could be construed as a potential conflict of interest.
